# Purification of protein C from canine plasma

**DOI:** 10.1186/s12917-014-0251-2

**Published:** 2014-10-18

**Authors:** Valerie M Wong, Dorothee Bienzle, M Anthony Hayes, Paul Taylor, R Darren Wood

**Affiliations:** Department of Pathobiology, Ontario Veterinary College, University of Guelph, Guelph, ON N1G 2W1 Canada; College of Veterinary Medicine, Midwestern University, 19555 N. 59th Ave, Glendale, AZ 85308 USA; Advanced Protein Technology Centre, THe Hospital for Sick Children, Toronto, ON M5G 1X8 Canada

**Keywords:** Dog, Plasma protein, Anticoagulant

## Abstract

**Background:**

In order to characterize the functional properties of canine protein C (CnPC), the zymogen needs to be purified from plasma. The goals of this study were (1) to purify protein C from fresh frozen canine plasma by barium chloride and ammonium sulphate precipitation, followed by immunoaffinity chromatography using a monoclonal mouse antibody against human protein C (HPC4) and (2) to characterize this protein’s structure.

**Results:**

The purified protein contained three glycosylated forms of a heavy chain (~49 kDa) and a glycosylated light chain (~25 kDa). Tandem mass spectra of the peptides obtained following trypsin digestion and liquid chromatography identified this protein to be protein C (vitamin K-dependent protein C precursor, gi|62078422) with 100% probability. Three glycosylation sites (Asn139, Asn202, and Asn350) were identified by detection of peptides containing an N-linked glycosylation consensus sequon with a 3-dalton increase in mass following incubation of the protein with PNGase F in ^18^O-labeled water. Following incubation with Protac (a specific activator of protein C), the heavy chain showed a slight decrease in molecular size and amidolytic activity measured by a synthetic chromogenic substrate containing an amide bond [H-D-(γ-carbobenzoxyl)-lysyl-prolyl-arginine-paranitroanilide diacetate salt]. The amidolytic activity was increased by ~303-fold in the final protein preparation compared to that in plasma. The purified protein showed concentration-dependent anti-factor V and anti-factor VIII activities in canine plasma in coagulometric factor assays.

**Conclusions:**

These studies showed that CnPC could be purified from plasma using HPC4 and that this protein showed amidolytic and anti-coagulant properties upon activation with Protac.

## Background

Activated protein C (APC) is a natural anticoagulant in plasma. It is derived from protein C zymogen, which is synthesized by hepatocytes and circulates in plasma at low concentration. Upon activation by thrombin, APC inhibits factor V (FV) and factor VIII (FVIII) activities by irreversible proteolysis [1,2]. Recombinant human activated protein C (rhAPC) was previously available as drotrecogin alpha (activated) (Xigris, Eli Lilly & Company, Indianapolis, IN) and approved for treatment of sepsis in patients with high risk of death by the United States Food and Drug Administration (FDA) and European Medicines Agency (EMEA) based on results obtained from the Recombinant Human Activated Protein C Worldwide Evaluation in Severe Sepsis (PROWESS) study [3]. A subsequent trial, PROWESS-SHOCK, showed no difference in the 28-day or 90-day mortality rates between the Xigris and placebo groups [4]. Furthermore, a systematic review of six randomized clinical trials, including the two aforementioned studies, showed no efficacy of treatment with Xigris in sepsis [5]. The manufacturer of Xigris eventually announced the withdrawal of Xigris from the market worldwide on October 25, 2011 [6].

Despite the lack of clinical efficacy of Xigris for treating sepsis, the anti-inflammatory and cytoprotective effects of protein C are well established. It has also been shown that the anticoagulant effects of APC are not required for protecting septic mice against mortality [7]. An advantage of using non-coagulant APC variants, as opposed to the anticoagulant wild-type APC, in a clinical setting is reduced risk of bleeding, as the use of Xigris in human sepsis was associated with increased risk of serious bleeding [8]. Furthermore, some APC variants conferred a higher degree of cytoprotection than wild-type APC [9,10].

The overall structure of the gene encoding for canine protein C (CnPC) zymogen is very similar to that for humans, consisting of nine exons and conserved exon/intron boundaries [11]. Computational analysis of CnPC cDNA predicted that CnPC had 456 amino acids and was 72.1% similar to human protein C [11]. Furthermore, in naturally occurring sepsis for both species, reduced circulating protein C activities were noted and early increase in protein C levels during hospitalization were of positive prognostic value [12-16]. The biological properties of CnPC are not as well established as those for human protein C. Given the therapeutic potential of rhAPC and human APC variants, the therapeutic potential of CnPC or canine activated protein C (CnAPC) warrants investigation. To facilitate such investigation, CnPC needs to be purified so that its precise structure and function may be determined. Knowledge of the CnPC pathway could potentially benefit canine patients and enhance our understanding of the protein C pathway in other species.

In this manuscript, we describe a method for purifying CnPC zymogen from fresh frozen plasma by salt precipitation followed by immunoaffinity chromatography using an antibody directed against a peptide sequence on human protein C (clone HPC4). This method was modified from a method described by Stenflo et al. for isolating protein C from fresh bovine plasma [17].

## Results

**Figure 1:**
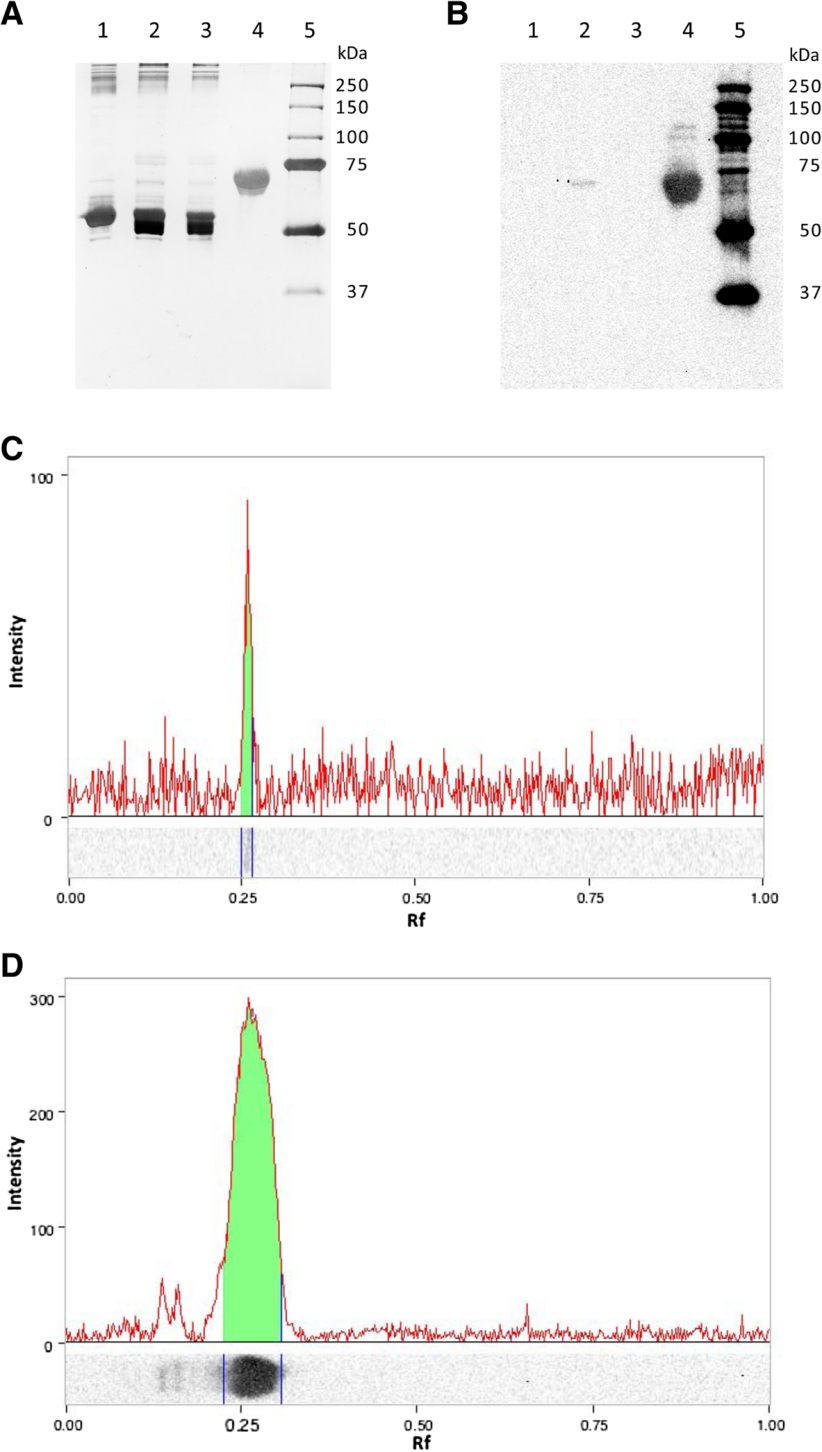
**Purification of CnPC from plasma by immunoaffinity chromatography using HPC4. (A)** Silver stained non-reduced 12% SDS-PAGE showing plasma protein fractions obtained over the course of purification. Five μg of protein was loaded into each lane. Lane 1: Whole plasma. Lane 2: Plasma protein fraction obtained after salt precipitation and exclusion of protein <30 kDa in size. Lane 3: Plasma protein fraction in the salt precipitate that did not bind to react with HPC4. Lane 4: Proteins obtained after salt precipitation and immunoaffinity purification. Proteins were ~75 kDa in size and migrated as two tightly spaced bands. Lane 5: Molecular weight markers. **(B)** Western blot of gel in **(A)** with antibody to peptide EDQVDPRLIDGK. Lane loads were the same as **(A)**. A faint band was present after salt precipitation and removal of small proteins (lane 2). This band was more prominent after immunoaffinity purification (lane 4). **(C)** Densitometry profile of lane 2 in **(B)**. **(D)** Densitometry profile of lane 4 in **(B)**. This band was 37.59 times as dense as the highlighted band in lane 2.

**Figure 2:**
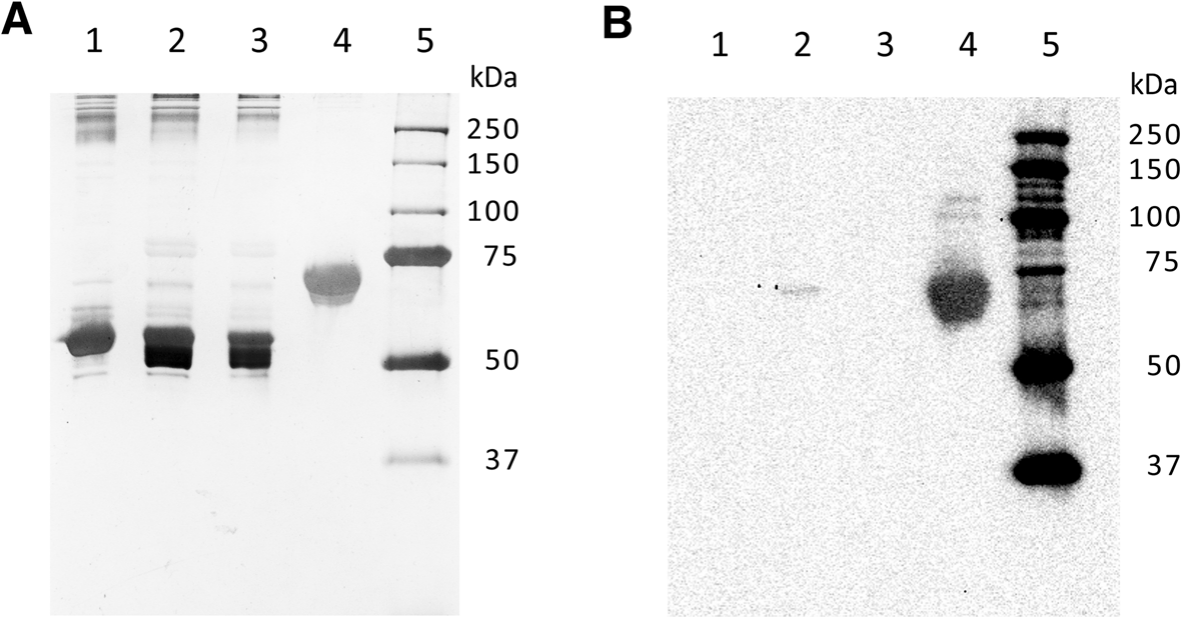
**A polyclonal anti-protein C antibody specifically detecting CnPC in plasma. (A)** Silver stained reducing 12% SDS-PAGE of human protein C-deficient plasma and with added CnPC. Lane 1: CnPC (0.5 μg) purified from canine plasma. Lane 2: Human protein C-deficient plasma (5 μg of protein). Lane 3: Human protein C-deficient plasma (5 μg of protein) spiked with 0.5 μg of CnPC. Lane 4: Molecular weight markers. **(B)** Western blot of gel in **(A)** probed with a polyclonal antibody to EDQVDPRLIDGK. Lane loads were the same as **(A)**. Two tightly spaced bands (likely two glycoforms) were present in lanes 1 and 3, indicating detection of CnPC. Only the major glycoform was visible in lane 1 in **(A)**, and this was likely related to lower sensitivity of silver staining than western immunoblotting.

A plasma protein was markedly enriched over the course of the purification process, which consisted of two major parts: salt precipitation (barium sulphate precipitation followed by ammonium sulphate precipitation) and immunoaffinity chromatography using a monoclonal antibody that targets the amino acid sequence EDQVDPRLIDGK of human protein C. On a silver-stained non-reducing sodium dodecyl sulphate polyacrylamide gel electrophoresis (SDS-PAGE) of the different plasma protein fractions obtained at various stages of the purification process, this protein was not readily discernable until after immunoaffinity chromatography (Figure 1A). However, Western immunoblotting with a polyclonal antibody detected this protein in small quantities in the plasma protein fraction obtained after salt precipitation and in much greater (37.6-fold increase) amounts in the eluate obtained from immunoaffinity chromatography (Figure 1B-D). This protein showed strong immunoreactivity for a polyclonal antibody against human protein C, while no other plasma proteins in human protein C-deficient plasma or canine plasma showed such immunoreactivity (Figure 2). Furthermore, using domain-enhanced lookup time-accelerated BLAST (DELTA-BLAST) [18] to search the Swissprot database for *canis lupus familiaris*, only CnPC (accession number Q28278.2) was found to show significant alignment with the sequence EDQVDPRLIDGK.

**Figure 3:**
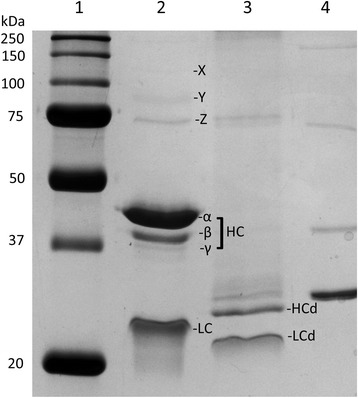
**Silver-stained reducing SDS-PAGE of CnPC before and after deglycosylation.** Lane 1: Molecular weight markers. Lane 2: Protein C purified from canine plasma consists of a heavy chain (HC) with three glycosylated forms (α, β, and γ) and a glycosylated light chain (LC). Lane 3: The same preparation shown in lane 2 treated with deglycosylation enzyme mixture. The heavy chain had decreased MW and migrated as a single band (HCd), suggesting CnPC has different glycosylated forms of the same peptide. Light chain also had decreased MW after deglycosylation (LCd). Lane 4: The enzyme mixture used in lane 3.

The peptide mass spectra obtained following in-gel trypsin digestion and liquid chromatography followed by tandem mass spectral analysis (LC-MS/MS) identified protein C (vitamin K-dependent protein C precursor, gi|62078422) in all silver-stained protein bands with 100% probability. Table 1 shows a summary of the spectra assigned to identification of protein C in each protein band. Table 2 shows a summary of the mass spectral data for each excised band. Only protein C was identified in the three closely spaced bands seen at ~49 kDa (MW of the three bands were estimated to be: 49 kDa, 44 kDa, and 40 kDa) and a band at ~25 kDa (Figure 3). Protein C was also identified in other bands (~129 kDa, ~107 kDa, and ~75 kDa) (Figure 3). The 75-kDa band may represent the single chain form of protein C, which is also found in human plasma. However, Western immunoblotting using antibodies against human protein C did not detect immunoreactivity in the 75-kDa region in reduced CnPC (Figure 2B). No further studies were performed to assess whether or not single-chain form of protein C was in canine plasma. Apolipoprotein A-1 was present in a band at ~25 kDa. Complement C3 was present in two bands at ~129 kDa and ~75 kDa.

**Table 1:** **Peptide sequences from LC-MS/MS confirming identify of protein C**

**Band**	**Sequence**	**Probability**	**Ion score**	**Identity score**
**HCα**	(R)DAcEGDSGGPMVTSFR(G)	95%	62.74	25.00
	(R)DAcEGDSGGPmVTSFR(G)	95%	77.48	25.00
	(R)eLTQVGQETVVTGWGYR(S)	95%	50.45	31.31
	(R)ELTQVGQETVVTGWGYR(S)	95%	39.26	31.38
	(R)ELTQVGQETVVTGWGYR(S)	95%	47.03	31.38
	(R)ELTQVGQETVVTGWGYR(S)	95%	70.81	31.38
	(K)GEmDVDIK(E)	95%	49.08	27.51
	(R)GESPWQVVLLDSK(K)	95%	71.42	30.85
	(R)GESPWQVVLLDSKK(K)	95%	30.54	30.09
	(R)GESPWQVVLLDSKK(K)	95%	63.18	30.09
	(R)GTWFLVGLVSWGEGcGR(L)	95%	67.5	31.20
	(R)GTWFLVGLVSWGEGcGR(L)	95%	59.65	31.20
	(R)HcScAPGYR(L)	95%	30.75	25.00
	(R)LGEYDLR(R)	95%	37.33	28.93
	(R)LGEYDLR(R)	95%	37.66	28.70
	(R)LGEYDLR(R)	95%	38.07	28.93
	(R)LGEYDLR(R)	95%	41.16	28.70
	(R)LVNGKVTR(R)	95%	51.78	27.82
	(R)RGESPWQVVLLDSK(K)	95%	40.51	30.07
	(R)RGESPWQVVLLDSK(K)	95%	41.43	30.25
	(R)RGESPWQVVLLDSK(K)	95%	41.91	30.07
	(R)RGESPWQVVLLDSK(K)	95%	52.68	30.25
	(R)RGESPWQVVLLDSK(K)	95%	45.81	30.22
	(R)RGESPWQVVLLDSKK(K)	95%	54.73	28.60
	(R)RGESPWQVVLLDSKK(K)	95%	55.67	28.61
	(K)STTDNDIALLHLAQPAIFSQTIVPIcLPDSGLAER(E)	95%	58.73	26.59
	(R)YLDWIHSHIR(G)	95%	38.67	30.04
	(R)YLDWIHSHIR(G)	95%	42.28	30.12
	(R)YLDWIHSHIR(G)	95%	43.78	30.12
	(R)YLDWIHSHIR(G)	95%	33.04	30.12
**HCβ**	(R)DAcEGDSGGPmVTSFR(G)	95%	82.07	25.00
	(R)DTNQTDQIDPR(L)	95%	43.85	27.71
	(R)DTNQTDQIDPR(L)	95%	44.08	27.76
	(R)DTNQTDQIDPR(L)	95%	43.09	26.50
	(R)ELTQVGQETVVTGWGYR(S)	95%	37.28	31.21
	(R)eLTQVGQETVVTGWGYR(S)	95%	40.06	31.36
	(R)eLTQVGQETVVTGWGYR(S)	95%	57.46	31.32
	(R)ELTQVGQETVVTGWGYR(S)	95%	45.45	31.38
	(R)ELTQVGQETVVTGWGYR(S)	95%	72.84	31.35
	(R)ELTQVGQETVVTGWGYR(S)	95%	50.99	31.29
	(R)ELTQVGQETVVTGWGYR(S)	95%	82.28	31.46
	(R)eLTQVGQETVVTGWGYR(S)	95%	82.84	31.33
	(K)EVLIHPNYSK(S)	95%	38.95	29.96
	(K)EVLIHPNYSK(S)	95%	53.99	29.85
	(K)GEmDVDIK(E)	95%	48.54	27.81
	(R)GESPWQVVLLDSK(K)	95%	83.15	30.54
	(R)GTWFLVGLVSWGEGcGR(L)	95%	62.12	31.30
	(R)GTWFLVGLVSWGEGcGR(L)	95%	52.34	31.20
	(R)LGDDHLQcQPAVK(F)	95%	29.27	30.38
	(R)LGEYDLR(R)	95%	36.72	28.93
	(R)LGEYDLR(R)	95%	38.59	28.93
	(R)RGESPWQVVLLDSK(K)	95%	43.76	30.07
	(R)RGESPWQVVLLDSK(K)	95%	48.35	30.24
	(R)RGESPWQVVLLDSK(K)	95%	52.51	30.22
	(R)RGESPWQVVLLDSK(K)	95%	45.78	30.20
	(R)RGESPWQVVLLDSKK(K)	95%	56.17	28.62
	(R)YLDWIHSHIR(G)	95%	27.58	30.04
	(R)YLDWIHSHIR(G)	95%	45.55	29.51
**HCγ**	(R)DAcEGDSGGPmVTSFR(G)	95%	55.88	25.00
	(R)eLTQVGQETVVTGWGYR(S)	95%	74.58	31.37
	(R)ELTQVGQETVVTGWGYR(S)	95%	84.14	31.35
	(R)GEEASLENQVP(-)	95%	36.07	27.59
	(R)GESPWQVVLLDSK(K)	95%	54.73	30.81
	(R)GTWFLVGLVSWGEGcGR(L)	95%	44.16	31.21
	(R)GTWFLVGLVSWGEGcGR(L)	95%	71.82	31.35
	(R)LGEYDLR(R)	95%	39.46	28.78
	(R)RGESPWQVVLLDSK(K)	95%	48.08	30.23
	(R)RGESPWQVVLLDSK(K)	95%	44.88	30.07
	(R)YLDWIHSHIR(G)	95%	39.9	30.10
**LC**	(R)DTnQTDQIDPR(L)	95%	48.54	26.91
	(R)ELTQVGQETVVTGWGYR(S)	95%	36.27	31.38
	(R)ELTQVGQETVVTGWGYR(S)	95%	89.79	31.45
	(R)GTWFLVGLVSWGEGcGR(L)	95%	75.93	31.30
	(R)HcScAPGYR(L)	95%	33.4	25.00
	(R)HcScAPGYR(L)	95%	42.26	25.00
	(R)HcScAPGYR(L)	95%	45.5	25.00
	(R)LGEYDLR(R)	95%	39.5	28.78
	(R)RGESPWQVVLLDSK(K)	95%	47.81	30.23
	(R)RGESPWQVVLLDSK(K)	95%	53.34	30.22
**HCd**	(R)DAcEGDSGGPmVTSFR(G)	95%	81.66	25.00
	(R)DTnQTDQIDPR(L)	95%	42.7	26.58
	(R)ELTQVGQETVVTGWGYR(S)	95%	41.89	31.35
	(R)ELTQVGQETVVTGWGYR(S)	95%	85.33	31.30
	(R)eLTQVGQETVVTGWGYR(S)	95%	93.64	31.37
	(K)EVLIHPnYSK(S)	95%	39.21	30.08
	(K)EVLIHPnYSK(S)	95%	47.75	29.87
	(K)eVLIHPnYSK(S)	95%	57.75	28.94
	(K)EVLIHPnYSK(S)	95%	60.43	30.12
	(R)GESPWQVVLLDSK(K)	95%	67.05	30.81
	(R)GTWFLVGLVSWGEGcGR(L)	95%	69.72	31.25
	(R)HcScAPGYR(L)	95%	29.11	25.00
	(R)RGESPWQVVLLDSK(K)	95%	39.91	30.23
	(R)RGESPWQVVLLDSK(K)	95%	39.82	30.23
	(R)RGESPWQVVLLDSKK(K)	95%	54.93	28.60
	(R)YLDWIHSHIR(G)	95%	30.81	30.12
	(R)YLDWIHSHIR(G)	95%	43.69	30.11
**LCd**	(R)ELTQVGQETVVTGWGYR(S)	95%	32.58	31.36
	(R)ELTQVGQETVVTGWGYR(S)	95%	85.2	31.30
	(R)HcScAPGYR(L)	95%	31.91	25.00
	(R)HcScAPGYR(L)	95%	32.88	25.00
	(R)LGEYDLR(R)	95%	41.34	28.93
**X**	(R)DTNQTDQIDPR(L)	95%	33.05	27.71
	(R)ELTQVGQETVVTGWGYR(S)	95%	25.29	31.31
	(R)ELTQVGQETVVTGWGYR(S)	95%	90.91	31.38
	(R)GEEASLENQVP(-)	95%	34.55	27.73
	(R)GESPWQVVLLDSK(K)	95%	67.83	30.85
	(R)LGDDHLQcQPAVK(F)	95%	23.14	30.39
	(R)LGEYDLR(R)	95%	37.69	28.78
	(R)RGESPWQVVLLDSK(K)	95%	42.97	30.05
	(R)RGESPWQVVLLDSK(K)	95%	40.33	30.07
	(R)YLDWIHSHIR(G)	95%	39.48	29.71
**Y**	(R)ELTQVGQETVVTGWGYR(S)	95%	31.23	31.29
	(R)ELTQVGQETVVTGWGYR(S)	95%	67.41	31.30
	(R)HcScAPGYR(L)	95%	28.9	25.00
	(R)LGDDHLQcQPAVK(F)	95%	24.31	30.32
	(R)LGEYDLR(R)	95%	41.34	28.93
	(R)RGESPWQVVLLDSK(K)	95%	25.09	30.05
**Z**	(R)ELTQVGQETVVTGWGYR(S)	95%	31.01	31.35
	(R)ELTQVGQETVVTGWGYR(S)	95%	97.66	31.32
	(K)GEmDVDIK(E)	95%	38.24	27.82
	(R)GESPWQVVLLDSK(K)	95%	75.58	30.85
	(R)LGDDHLQcQPAVK(F)	95%	40.02	30.39
	(R)LGEYDLR(R)	95%	41.54	28.93
	(R)RGESPWQVVLLDSK(K)	95%	28.86	30.07
	(R)RGESPWQVVLLDSK(K)	95%	41.52	30.24
	(R)YLDWIHSHIR(G)	95%	25.66	30.11

Bands as shown in Figure 3. Letters in lower case indicate either oxidation of methionine (m), carbamidomethyl cysteine (c), deamidation of asparagine (n) or pyroglutamate (e).

**Table 2:** **Summary of LC-MS/MS results**

**Band**	**Vitamin K-dependent protein C precursor** ^**a**^	**Apolipoprotein A-1 (predicted)** ^**b**^	**Complement C3 (predicted)** ^**c**^	**Albumin** ^**d**^
**HCα**	30 (13,19), 30%			
**HCβ**	28 (12,16), 27%			
**HCϒ**	11 (8,10), 20%			
**LC**	10 (6,8), 16%			
**HCd**	17 (10,13), 23%			
**LCd**		7 (7,7), 30%		
**Band X**	10 (8,10), 18%		10 (23,24), 15%	24 (3,3), 6%
**Band Y**	6 (5,6), 13%			
**Band Z**	9 (7,9), 15%		12 (11,12), 9%	22 (18,21), 33%

Bands according to Figure 3. Entries denote number of assigned spectra (number of unique peptides, number of unique spectra), percent coverage. Only proteins with peptide probability of ≥95%, protein probability of ≥99.9%, and ≥5 accepted peptides are listed.

^a^NM_001013849.1.

^b^NC_006587.3.

^c^NC_006587.3.

^d^NM_001003026.1.

**Figure 4:**
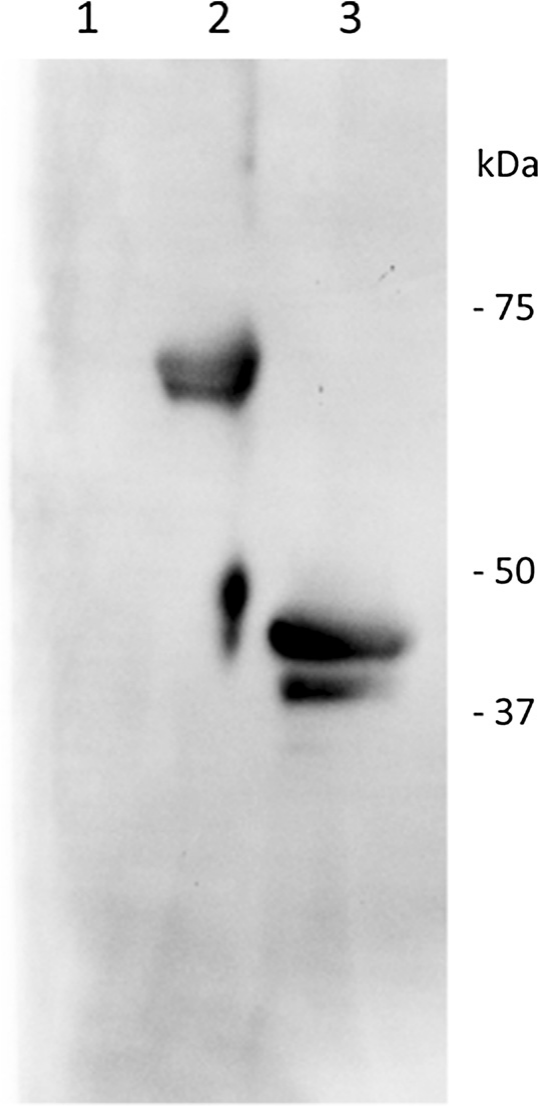
**Canine protein C was not a single-chain polypeptide.** Western blot of 12% SDS-PAGE using GMA-093 (which targets the heavy chain of human protein C) depicting CnPC purified from canine plasma by salt precipitation and immunoaffinity chromatography. Lane 1: Unbound protein fraction from immunoaffinity chromatography did not contain protein C. Lane 2: Five μg of non-reduced CnPC purified from canine plasma. Immunoreactivity was detected at ~75 kDa. Lane 3: Five μg of reduced CnPC purified from canine plasma. Immunoreactivity was detected at ~49 kDa due to dissociation from the light chain.

**Figure 5:**
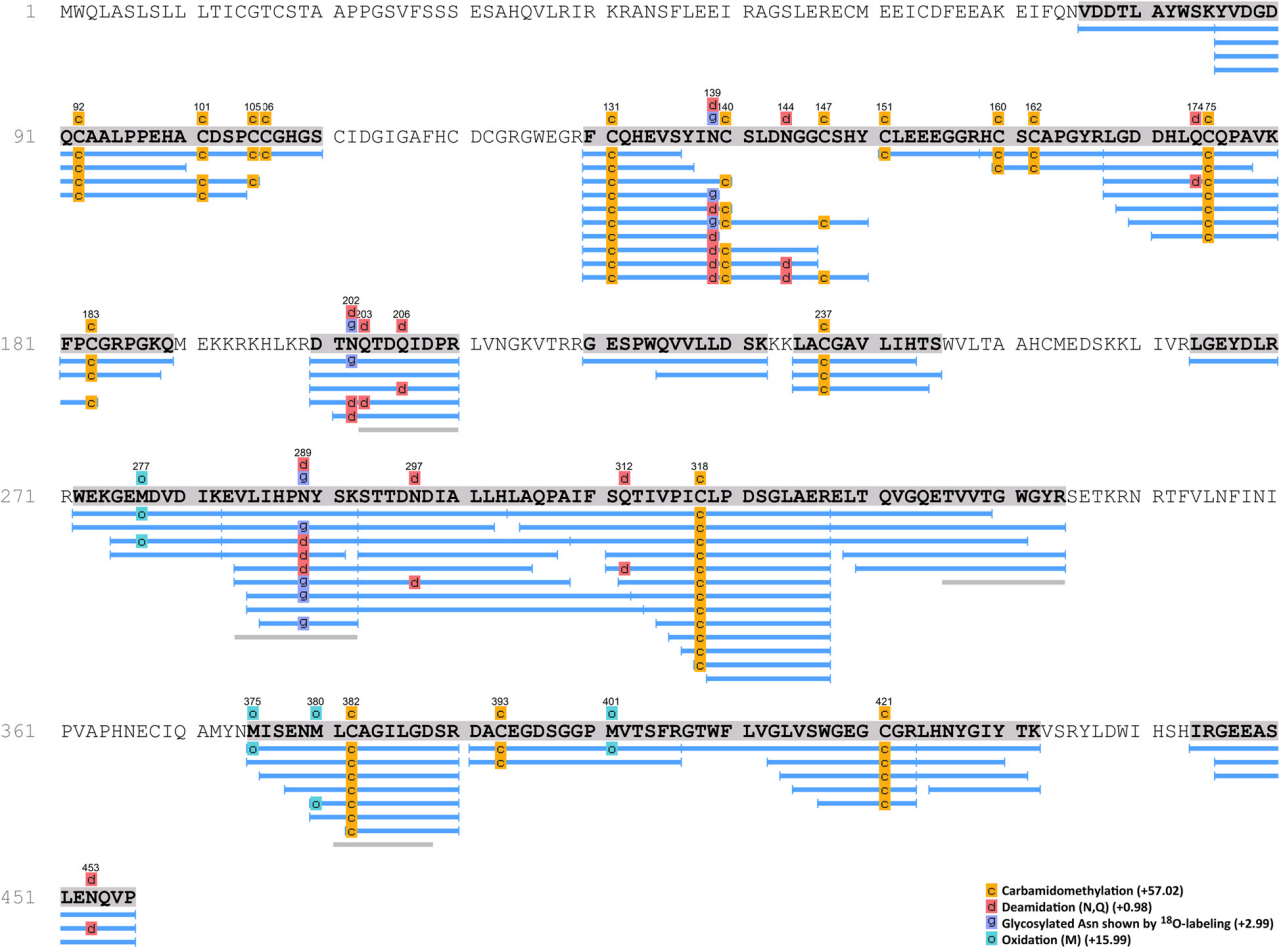
**Peptides derived from purified CnPC deglycosylated with PNGaseF in** ^**18**^ **O-labeled water.** The complete sequence was predicted from cDNA amplified by Leebs et al. and confirmed in our lab. Fully identified peptides are indicated in blue; peptides with matching sequences but incorrect masses are indicated in grey. Three (Asn139, Asn202, and Asn289) of the potential N-linked glycosylated sites showed a 3-dalton increase in mass, consistent with deamidation of the asparagine residue and incorporation of an ^18^O atom. The fourth potential N-linked glycosylated site (Asn350) was not captured on mass spectrometry on multiple attempts. Therefore, the glycosylation status of this site remains unknown. Natural deamidation (i.e. 1-dalton increase in mass at sites with asparagine that is not part of the N-X-S/T sequon or glutamine) was rare.

**Figure 6:**
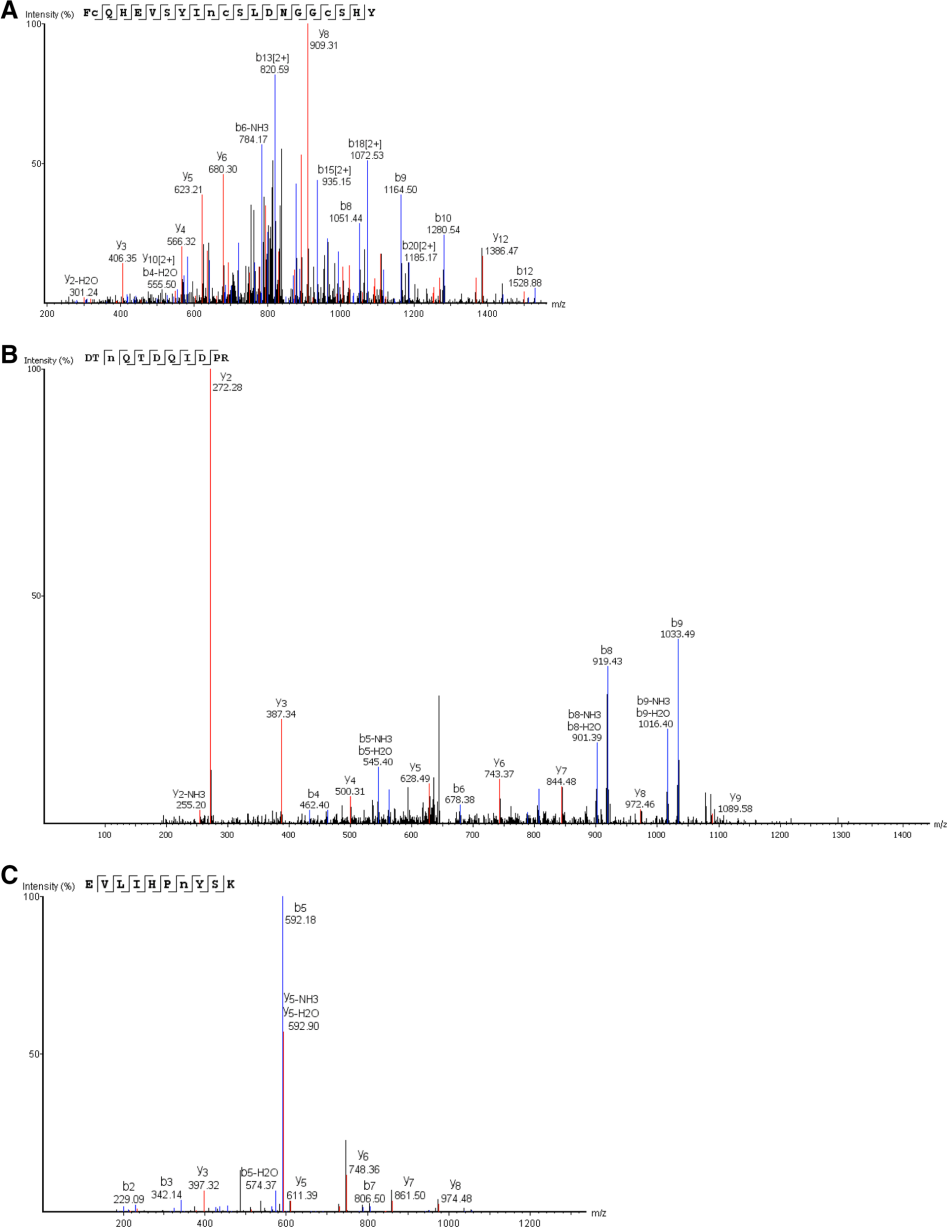
**N-linked glycosylation mapping by IGOT showing three N-linked glycosylated sites.** Tandem mass spectra of peptides showing a 3-dalton increase in mass at Asn139 **(A)**, Asn202 **(B)**, and Asn289 **(C)** after digestion with PNGase F in ^18^O-labeled water, suggesting that this site was previously glycosylated.

The structure of the purified protein showed many similarities with human protein C. The protein migrated as two tightly spaced bands of ~75 kDa under non-reducing conditions (Figures 1 and 4); and as three tightly spaced bands of ~49 kDa and a much smaller band of ~25 kDa (Figure 2). The results suggest that the protein consisted of two chains linked by at least one disulphide bond. To show the tightly spaced bands were different glycosylated forms, as opposed to different peptides or different degraded forms of the same peptide, the protein was reduced and then deglycosylated in a mixture of deglycosylation enzymes. The protein was first reduced to allow better access of the enzymes to their substrates. Following glycosylation, the three tightly spaced bands seen at ~49 kDa were no longer detected on SDS-PAGE (12%, silver-stained) (Figure 3). A smaller, single band was seen at ~27 kDa instead, suggesting that the three tightly spaced bands were indeed different glycosyated forms of the same peptide. The 25-kDa band that was seen with the parent form of the protein was also no longer detectable after deglycosylation. Instead, a new, smaller band was seen at ~23 kDa, suggesting that the parent form of this peptide was glycosylated as well (Figure 3). To determine the exact N-linked glycosylated sites of this protein, we searched the amino acid sequence of the protein for the consensus sequences (also known as sequons) N-X-T and N-X-S. The following four sites were identified: Asn139, Asn202, Asn289, and Asn350. It is recognized that these sequons are not the only requisite for N-linked glycosylation to occur [19,20]. To confirm N-linked glycosylation at these four sites, isotope-coded glycosylation site-specific tagging (IGOT) [21] was performed. This method is based on the principle that while PNGase F removes a glycan from the Asn residue, it deamidates Asn, converting it into an Asp residue. During this process, an oxygen ion from a water molecule is incorporated into the new Asp residue, which makes it 1 dalton heavier than the predicted Asn residue. Given that natural deamidation of Asn residues is known to occur, a more definitive way to show that deamidation is due to the action of PNGase F is to incorporate a labeled oxygen ion, such as ^18^O-labelled water. Deamidation of Asn ^18^O-labelled water will result in incoporation of an oxygen ion that is 2 daltons heavier than the H_2_^16^O, which is the more abundant form found in nature. This reaction will result in a 3-dalton mass shift, which is very unlikely to be a natural occurrence. By use of IGOT, three of the potential N-linked glycosylated sites were found to show a 3-dalton increase in mass: Asn139, Asn202, and Asn289 (Figures 5 and 6). The fourth N-linked glycosylated site (Asn350) was not captured on mass spectrometry on multiple attempts. Therefore, the glycosylation status of this site remains unknown.

**Figure 7:**
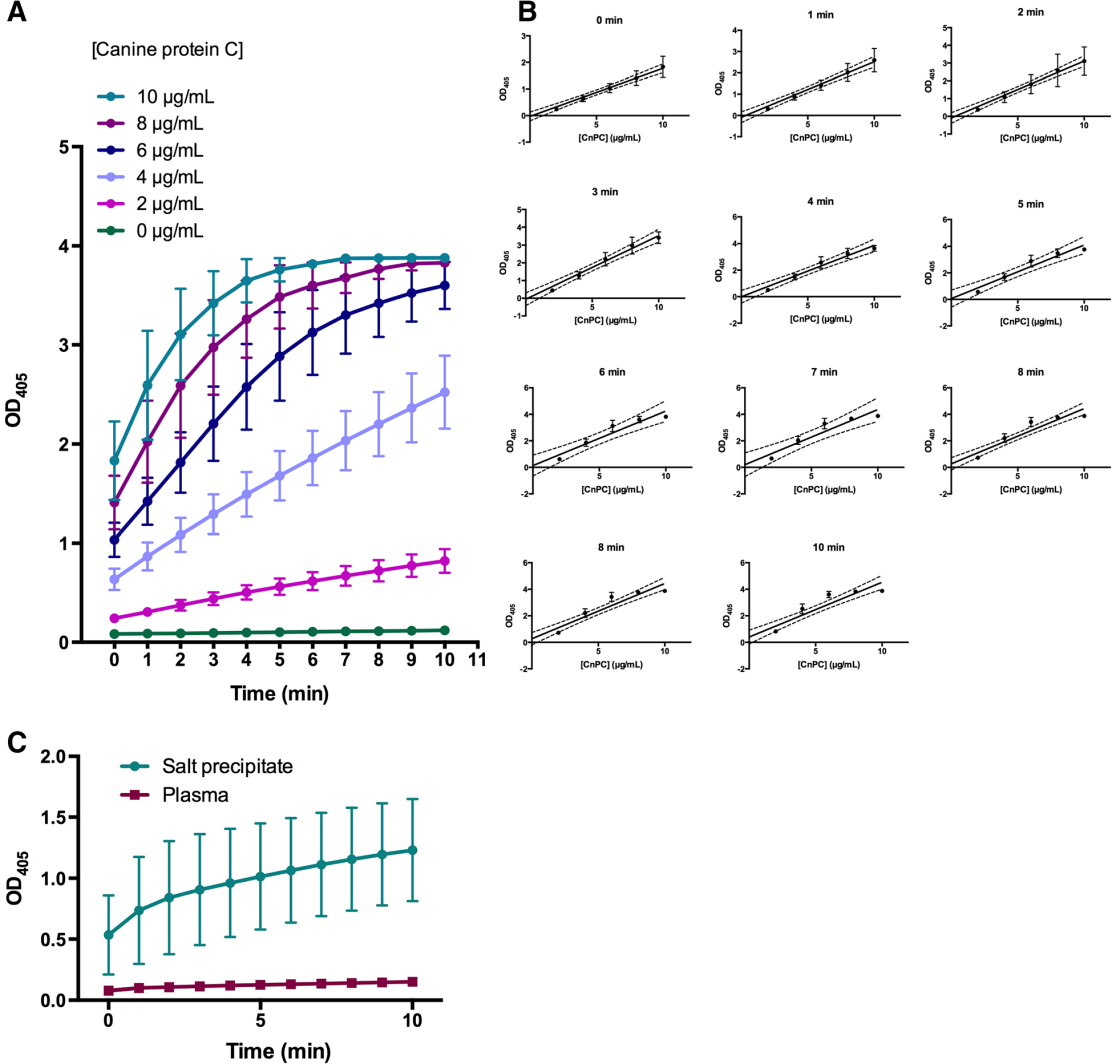
**Activation of CnPC by Protac, followed by detection of amidolytic activities with Spectrozyme.** All measurements were performed in duplicate, and the average of the two measurements was used as one true experimental replicate. Activity levels were expressed in optical density units measured at wavelength =405 nm. Each data point shown is the mean (±SEM) of three experiments using plasma from three different dogs. **(A)** Amidolytic activities detected at ten time points following addition of Spectrozyme. These results show that purified CnPC was activatable and retained its amidolytic activities. **(B)** Linear regression analysis depicting the relationship between the amount of amidolytic activities detected (expressed by optical density measured at wavelenghth =405 nm) and concentration of CnPC present at various time points. These results show that the linear range of 0–10 μg/mL in this assay was valid if the samples were analyzed within 5 minutes following addition of Spectrozyme. Each point is the mean (±SEM) of three experiments using plasma from three different dogs. The dotted lines delineate the 95% confidence intervals for the curves. **(C)** Activation of protein C in canine plasma and a plasma fraction obtained following barium chloride and ammonium sulphate precipitation. Final total protein concentration was adjusted to 100 ng/mL. Protein C was activated by Protac, a specific activator of protein C, followed by detection with the chromogenic substrate Spectrozyme. Measurements were taken at ten time points following addition of Spectrozyme in the system.

Both human [22,23] and CnPC [12,13,24] are known to show amidolytic activity following incubation with Protac. For human protein C, Protac removes a peptide from the heavy chain, thereby exposing the catalytic site, which cleaves FV and FVIII, as well as the synthetic chromogenic substrate Spectrozyme. To show that the protein purified in this study shared these properties, we investigated its amidolytic activity in a plasma-free system and effects on FV and FVIII activities in canine plasma. Following incubation with Protac, the protein showed amidolytic activities that were directly proportional to the protein concentration (Figure 7A and B). Such activities appeared to be enriched during the purification process as well. Initial attempts to detect protein C amidolytic activities in plasma and the salt precipitate using final protein concentrations of 10 μg/mL did not yield consistently detectable amidolytic activities. Activity was only detected when the final protein concentration was increased to 100 μg/mL (Figure 7C). Compared to plasma, the amidolytic activities of CnPC was increased by ~8.19-fold after salt precipitation and (after adjustment for the final protein concentration used in the assay) ~303-fold following immunoaffinity chromtagraphy (i.e. CnPC activity was enriched ~37.6 times in the final purified form compared to that in the salt precipitate). These results corroborated with results obtained by western immunoblotting using anti-protein C tag antibody.

**Figure 8:**
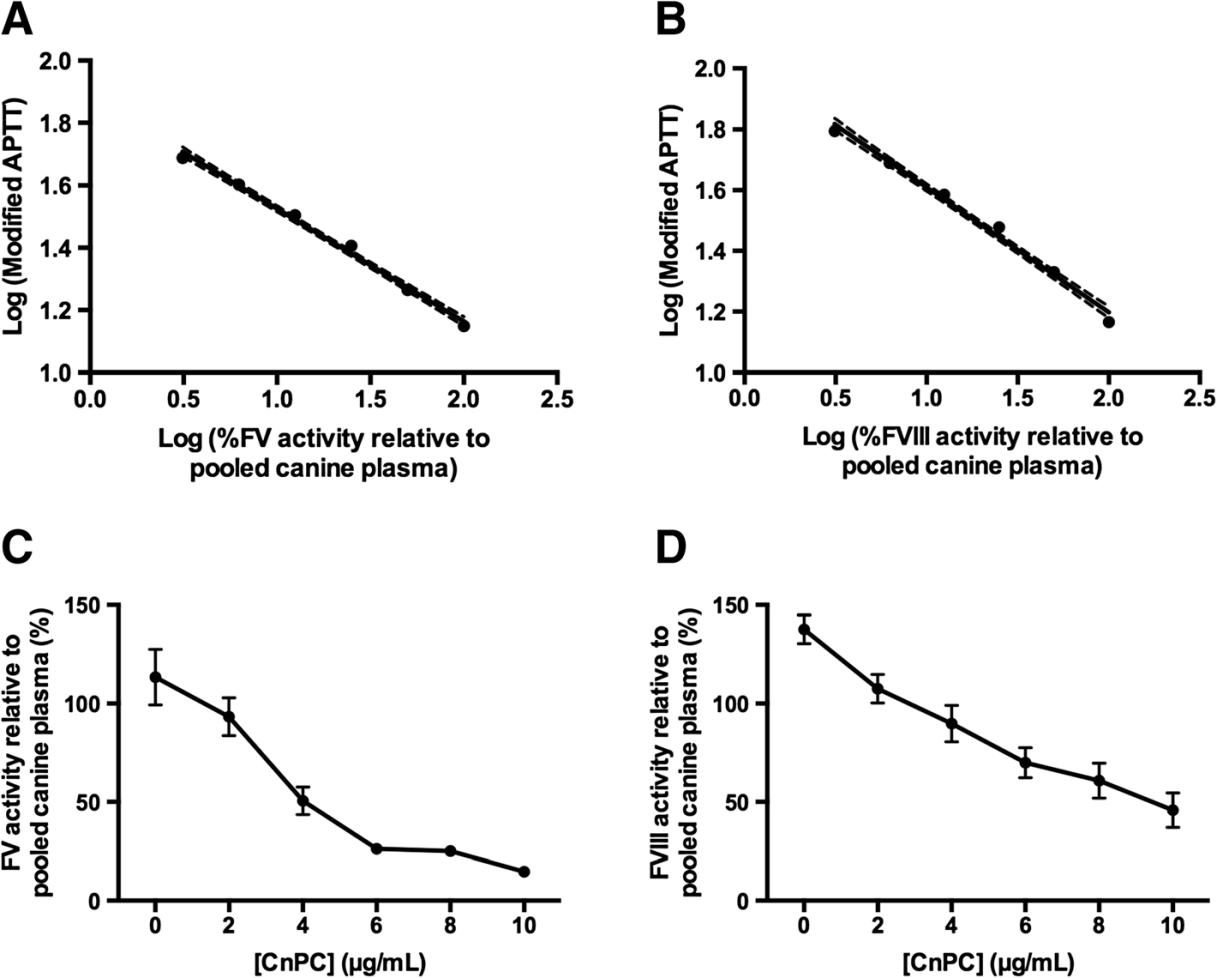
**The effects of purified CnPC on the activities of FV and FVIII in canine plasma.** Canine plasma pooled from ten dogs and used as a calibrator. For calibration **(A and B)**, the activity level of each coagulation factor in undiluted plasma pool was designated to be 100%; and five 2-fold dilutions yielded activity levels of 50%, 25%, 12.5%, 6.25%, and 3.125%. The activity level of each factor was expressed as a modified APTT, which reflected the abilities of the calibrators to correct the APTT in plasma deficient of a specific coagulation factor. All samples were analyzed in duplicate and the average of the two measurements was used as one true replicate for statistical analysis. Each data point represents the mean (±SEM) of five replicates. The modified APTTs generated in the presence of activated CnPC were compared against a calibration curve generated within the same assay. Percent activity of FV or FVIII was plotted against concentration of the purified canine protein **(C and D)**. Each data point depicted represents the mean (±SEM) of five replicates. The dotted lines delinate the 95% confidence intervals for the curves generated by linear regression.

The preservation of amidolytic activities of the purified protein suggested to us that the protein would most likely retain its anticoagulant properties as well. To confirm this, the effects of this protein (following activation in vitro by Protac) on the activities of FV and FVIII in canine plasma were determined. As predicted, activated form of the protein inhibited the activities of these coagulation factors in a concentration-dependent manner (Figure 8).

**Figure 9:**
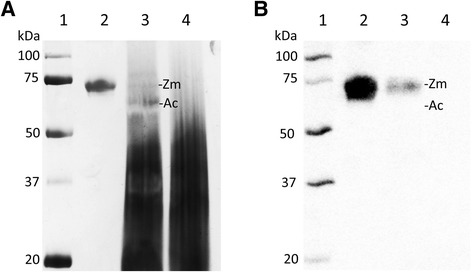
**Digestion of CnPC with Protac. (A)** Silver stained non-reduced 12% SDS-PAGE depicting the plasma protein fractions obtained over the course of purification of protein C from canine plasma. Five μg of protein C sample (zymogen or activated) was loaded into each lane. Lane 1: Molecular weight markers (3 μL). Lane 2: CnPC purified from canine plasma. Lane 3: CnPC following incubation with Protac, a specific activator of protein C; note the decrease in MW of activated CnPC (Ac) and small amount of undigested zymogen form (Zm). Lane 4: Protac purchased commercially. **(B)** Western blot of the adjacent SDS-PAGE (A) with a polyclonal antibody against the peptide EDQVDPRLIDGK found in human protein C zymogen, but not in the activated form of protein C. Lane loads were the same as **(A)**. An immunoreactive protein fraction was detected in the undigested sample. Following incubation with Protac, the amount of immunoreactive protein (Zm) was significantly decreased. The activated form of the protein (Ac) was, as predicted, not immunoreactive to the primary antibody.

Other researchers have concluded that human and CnPC share structural similarities when comparing the expression sequences between the two species [11]. More specifically, they predicted that CnPC was synthesized as a single-chain protein, which was processed into a double-chain protein by removal of a dipeptide adjoining the two chains, accompanied by removal of a signal peptide at the N-terminus. We performed N-terminal sequencing of the light and heavy chains of the purified protein, as well as the Protac-activated heavy chain to determine if findings at the protein level would corroborate with predictions based on alignment of the canine and human expression sequences. Edman sequencing showed that the N-terminus of the light chain began as: ANSFL, and that of the heavy chain began as: DTXQT, where X could be a modified amino acid (a glycosylated asparagine in this case). These results corroborated with the predictions. Following incubation with Protac, CnPC showed a decrease in molecular weight (Figure 9), and the N-terminus of the heavy chain was determined to be GVNGK by Edman sequencing. However, CnPC cDNA obtained by us (data not shown) and others [11,25] showed that the amino acid residue prior to VNGK should be a leucine, and LC-MS/MS results showed that this amino acid at this position in the peptide had a mass consistent with either leucine or isoleucine (the only two natural amino acids that share the exact same mass). We concluded that the N-terminal sequence of Protac-digested heavy chain was likely LVNGK, and the glycine detected was probably from a second protein or peptide present in the sample.

## Discussion

In this study, we showed that a method involving salt precipitation followed by immunoaffinity chromatography using a monoclonal antibody directed against human protein C zymogen (clone HPC4) was useful for purifying protein C zymogen from canine fresh frozen plasma. This method is straightforward and does not require expensive equipment. The matrix and antibody are coupled as a commercially available product, which can be used up to 7 to 10 times, according to the manufacturer’s instructions, without the need for regeneration. The proteins purified from fresh frozen plasma using this method were identified to be CnPC based on tandem mass spectrometry and western immunoblotting. Using a method that quantified the amidolytic activity of the activated form of the protein, we showed that such activity was enriched 8.19-fold by salt precipitation and a further 37.59-fold by immunoaffinity chromatography. A specific antigenic assay for CnPC was not available at the time of this study, rendering absolute quantification of the yield and purification level of the final protein preparation impossible.

Clone HPC4 targets the peptide sequence EDQVDPRLIDGK in human protein C zymogen [26], and the corresponding sequence in CnPC, TDQIDPRLVNGK, differs at four of twelve amino acid residues. Despite these differences, HPC4 showed sufficient cross-reactivity with CnPC for protein purification and western immunoblotting purposes. It has been suggested that calcium ions expose the HPC4-binding site by inducing a conformational change in the protein [26]. Calcium ions may induce a conformational change in CnPC such that differences in primary amino acid sequence do not affect antibody binding.

In humans, protein C is produced by hepatocytes as a single-chain protein [27]. During post-translational modification (PTM), the pre-pro-peptide at the N-terminus is removed [28]. Additionally, an internal arginine-lysine dipeptide is removed, resulting in a heavy chain and light chain linked by a disulphide bond [29]. By performing densitometry on Western blots, Greffe et al. showed that in human adults, 90% of the plasma protein C was double-chained, while 10% was single-chained [30]. They also showed that single-chain human protein C had three isoforms: alpha (MW 62 kDa), beta (MW 58 kDa), and gamma (MW 55 kDa); that the heavy chain had three isoforms alpha (MW 41 kDa), beta (MW 37 kDa), and gamma (MW 34 kDa); and that the light chain had two isoforms (MW 21 kDa and MW 18 kDa).

Our findings show that, similar to human protein C, CnPC exists in different glycoforms as well. On reducing SDS-PAGE, CnPC showed three distinct bands of MW 49 kDa, 44 kDa, and 40 kDa. These bands likely represent three different glycosylated forms of the heavy chain in CnPC, as treatment with a mixture of deglycosylation enzymes reduced these three bands into one single band and resulted in a decreased in MW. A faint band was visible at ~75-kDa; this may represent the single-chain CnPC. Results from LC-MS/MS analysis did confirm the presence of protein C in this band, and no immunoreactivity with HPC4 was detected in this region on western immnoblotting of the reduced form of the protein. We concluded that further studies were necessary to confirm the presence of single-chain CnPC in plasma.

We further determined three N-linked glycosylation sites on the CnPC. However, no further studies were performed to determine the biological significance of the glycans present at these sites. In humans, glycans on protein C appear to affect the functional properties of activated protein C, and the effect of glycosylation at Asn329 is best described among all the N-linked glycosylated sites. This site is conserved across many mammalian species [10]. Compared to wild-type APC, a recombinant APC variant with the glycan eliminated at Asn329 (ACP-N329Q) showed enhanced endothelial barrier protective function by 6-fold and improved anti-apoptotic function by 30-fold in endothelial cells treated with staurosporine. The effects of Asn329 on anticoagulation are not as well defined. APC-N239Q increased the anticoagulant effects [10,31], but naturally occurring protein C with a mutation at this site (N329T) showed decreased rate in deactivation of FVa following purification from plasma [32].

Canine protein C isolated by the method described here not only preserved the immunogenic properties of the protein, as demonstrated by Western immunoblotting, but also the amidolytic and anticoagulant properties. A commercially available protein C assay has been validated for measuring protein C activity in canine plasma [24]. This assay is based on the amidolytic activity of activated protein C on a chromogenic substrate, which yields a quantifiable change in absorbance upon cleavage by activated protein C. In this study, we showed that CnPC purified by the described method was activatable by Protac and retained its amidolytic properties. Similarly, following *ex vivo* activation with Protac, CnPC inhibited FV and FVIII activities in a dose-dependent manner, as expected. It is known that human APC inhibits FV and FVIII activities by proteolysis of these factors [1,2]. The same mechanisms are presumably present in dogs. Compared to human protein C, relatively little is known about CnPC.

Currently, the main clinical application of protein C in veterinary medicine is the differentiation of microvascular dysplasia and portosystemic vascular anomaly in dogs [33]. Combined with other clinical and laboratory data, plasma protein C activity could be used to distinguish portosystemic vascular anomaly from microvascular dysplasia [33]. Although not routinely performed, plasma protein C levels were found to have prognostic value in canine sepsis [13]. Clinical application of purified CnPC is not shown in this manuscript or described elsewhere and warrants further investigation.

## Conclusions

We showed that a functional protein C zymogen could be purified by salt precipitation followed by immunoaffinity chromatography using a commercially available matrix coupled to an antibody against human protein C. Upon activation with Protac, the purified protein retained its amidolyic, anti-FV, and anti-FVIII properties. We concluded that this method was useful for purifying CnPC for future functional studies.

## Methods

### Sources of CnPC

All procedures were carried out with the approval of the Animal Care Committee of the University of Guelph and in compliance with the guidelines provided by the Canadian Council on Animal Care, and informed client consent was obtained. Healthy adult dogs were selected for blood donation based on clinical history, physical examination, routine blood testing that included complete blood count and biochemistry, urinalysis, and testing for heartworm, erhrlichiosis, Lyme disease, anaplasmosis, and hemoplasmosis. Approximately 450 mL of whole blood was collected aseptically by a trained veterinary technician from the jugular vein into a bag containing citrate-phosphate-dextrose and Optisol (sodium, dextrose, adenine, and mannitol) (Teruflex Blood Bag, Terumo, Somerset, NJ) with constant mixing during collection. The bag was immediately centrifuged at 5,000 g at 4°C for 8 min, followed by immediate separation of the plasma from other blood components into the attached satellite bag. The plasma was stored at -20°C for up to one year, after which the fresh frozen plasma was moved to storage at -70°C for up to two years.

### Salt precipitation

Unless specified, all procedures described below, including centrifugation, were performed at 4°C. All reagents and containers were pre-chilled. Where protease inhibitors were used, they were prepared by dissolving tablets containing a mixture of protease inhibitors (Complete, EDTA-free, Protease Inhibitor Cocktail Tablets, Roche, Laval, QC) in the solution specified in each of the following steps described below so that the final concentration during incubation, centrifugation, or washing was one tablet per 50 mL of solution, as suggested by the manufacturer.

A bag of fresh frozen plasma was thawed in a cool water bath and divided into 40-mL aliquots in 50-mL BD Falcon polypropylene conical tubes (BD Biosciences, Mississauga, ON). 3.2 mL of 1 M barium chloride (Fisher Scientific, Ottawa, ON) containing protease inhibitors was added one drop at a time to each aliquot with periodic mixing. The plasma mixture was allowed to equilibrate for an additional one hour with constant mixing, followed by centrifugation at 1,500 g for 15 min. The supernatant was discarded, and the precipitate was resuspended in 10 mL of 0.9% NaCl containing protease inhibitors. This plasma protein fraction was centrifuged at 1,500 g for 15 min. After two such washes, the precipitate from two of the original 40-mL plasma aliquots was resuspended in 10 mL of 0.2% EDTA containing protease inhibitors. This suspension was allowed to equilibrate for 30 min with constant mixing. Ammonium sulphate pellets (Fisher Scientific, Ottawa, ON) were added to the suspension in small quantities with periodic mixing to achieve a final saturation level of 40% (243 mg per mL solution). This was followed by 10 min of constant mixing to ensure complete solubilization of the ammonium sulphate pellets and equilibration. The suspension was centrifuged at 1,500 g for 30 min. The supernatant was separated. Ammonium sulphate pellets were added in the same manner described above to achieve a final saturation level of 65% (168 mg per mL solution). This was followed by 10 min of constant mixing and then centrifugation at 1,500 g for 30 min. The supernatant was removed. The precipitate was dissolved in 2 mL of water. A buffer exchange into 20 mM Tris, pH 7.5, containing 0.1 M sodium chloride, 1 mM calcium chloride, and protease inhibitors was performed immediately using 15-mL Amicon Ultra filter units with molecular weight cut-off of 30 kDa (Millipore, Billerica, MA). After the final spin, the retentate was resuspended in the same buffer containing protease inhibitors so that proteins isolated from two of the original 40-mL plasma aliquots was resuspended in 1 mL of solution.

### Immunoaffinity chromatography

The following procedure was carried out at 4°C with chilled reagents and containers. The same protease inhibitor tablets were used in the same manner as described above. The matrix described in the following paragraphs refers to a commercially available product (Anti-Protein C Affinity Matrix; Roche, Laval, QC), which consists of agarose beads coupled to a monoclonal antibody (clone HPC4; mouse IgG1κ) against a 12-amino acid sequence (EDQVDPRLIDGK) of human protein C.

One mL of matrix was packed in the column by gravitation. The matrix was equilibrated with 10 bed-volumes of equilibration buffer (20 mM Tris, pH 7.5, containing 0.1 M sodium chloride, 1 mM calcium chloride). The buffer was drained. The bottom end of the column was capped and 1 mL of equilibration buffer containing protease inhibitors was applied to the matrix. One mL of the plasma protein suspension obtained after barium chloride and ammonium sulphate precipitation was applied to the matrix. The final concentration of calcium chloride was adjusted to 1 mM. The top of the column was capped. The column, now containing the proteins and the matrix, was inverted a few times to loosen the matrix, followed by gentle constant mixing on an end-to-end rocker for two hours. The matrix was then allowed to settle for 10 min. The bottom was uncapped and the unbound protein fraction was allowed to drain. A 23-g needle was applied to the bottom of the column. This set-up resulted in a flow rate of approximately 0.5 mL per minute. The matrix was washed with 30 bed-volumes of wash buffer (20 mM Tris, pH 7.5, containing 1.0 M sodium chloride, 1 mM calcium chloride) containing protease inhibitors. A clean cap was applied to the bottom of the column. Five mLs of elution buffer (20 mM Tris, pH 7.5, containing 0.1 M sodium chloride and 5 mM EDTA) was applied to the matrix, followed by 30 minutes of incubation without mixing. The bottom of the column was uncapped and the 5 mL of elution buffer, now containing the eluted proteins, was collected through a fresh 23-g needle. A buffer exchange into the equilibrium buffer was performed using 4-mL Amicon Ultra filter units with molecular weight cut-off of 30 kDa (Millipore, Billerica, MA). The retentate was concentrated to 250–500 μL in the last spin.

### SDS-PAGE

The eluted proteins were subjected to one-dimensional SDS-PAGE under both denaturing and non-denaturing conditions. Buffers were reconstituted from stock solutions (Bio-Rad, Mississauga, ON) for a modular Mini-Protean II system (Bio-Rad, Mississauga, ON). Laemmli sample buffer (Bio-Rad, Mississauga, ON) and β-mercaptoehthanol (Sigma-Aldrich, Oakville, ON) were purchased and used according to the manufacturer’s instructions. Briefly, 1 part β-mercaptoethanol was added to 9 parts of Laemmli sample buffer. For denaturing SDS-PAGE, 5 μg of proteins was combined with this sample buffer at a 1:1 (v:v) ratio, followed by incubation at 65°C for 10 minutes. For non-denaturing SDS-PAGE, 5 μg of eluted proteins was directly combined with the Laemmli sample buffer at a 1:1 (v:v) ratio and kept at room temperature prior to electrophoresis without addition of β-mercaptoethanol. The protein samples were loaded into sample wells of a 1.0-mm thick, 15% Tris-HCl polyacrylamide gel and then subjected to SDS-PAGE at 220 V on ice for 75 min. The gels were then stained with silver for visualization of protein bands. Briefly, the gels were fixed in a solution of 40% ethanol and 10% glacial acetic acid, followed by rinsing with 20% ethanol and then water. The gels were then incubated with 0.2 g/L of sodium thiosulfate for one min, followed by rinsing with water, and then incubation with 2 g/L of silver nitrate for 30 minutes. Following a brief rinse with water, the gels were developed in a solution containing 30 g/L of sodium carbonate, 1.4 mL/L of 37% formaldehyde, and 10 mg/L of sodium thiosulfate for up to 15 min until the desired intensity of the protein bands was achieved. Molecular weights of the visible bands were compared against markers of known molecular weight (Precision Plus Protein WesternC Standards, Bio-Rad, Mississauga, ON).

### Mass spectrometry and protein identification

The silver-stained protein bands visible on SDS-PAGE were excised, placed in siliconized microcentrifuge tubes containing 1% v/v acetic acid, and submitted on dry ice for LC-MS/MS (The Advanced Protein Technology Centre, Hospital for Sick Children, Toronto, ON). In-gel tryptic digestion was performed as previously described [34]. Briefly, the silver-stained gel incubated with a solution containing 30 mM of potassium ferricyanide and 100 mM of sodium thiosulfate at a 1:1 (v/v) ratio for 15 minutes, followed by rinsing with water and then with 50 mM of ammonium bicarbonate. The gel was shrunk with 50% acetonitrile containing 25 mM of ammonium bicarbonate for 10 min. The proteins were then reduced in 10 mM of dithiothreitol (DTT) at 56°C for 30 min, followed by alkylation in 100 mM of iodoacetamide for 15 min. The gel was shrunk for the second time for 15 minutes. The gel was incubated overnight with 50 mM ammonium bicarbonate containing 13 ng/μL of porcine trypsin (Sequencing Grade Modified Trypsin, Promega, Madison, WI) at 37°C. The digested peptides were loaded onto a 150 μm ID pre-column (Magic C18, Michrom Biosciences, Auburn, CA) at 4 μL per min and separated over a 75 μm ID analytical column packed into an emitter tip containing the same packing material. The peptides were eluted over 60 minutes at 400 nL per minute using a 0–40% acetonitrile gradient in 0.1% formic acid into a linear ion trap mass spectrometer (LTQ-Orbitrap) (Thermo-Fisher, San Jose, CA) operated in a data-dependent mode. One MS followed by 6 MS/MS scans were obtained per cycle. Tandem mass spectra were extracted by Extract_msn.exe 4.0 and Bioworks 3.3 (Thermo-Fisher, San Jose, CA). Charge state deconvolution and deisotoping were performed. The generated data files were analyzed using Mascot 2.3 (Matrix Science, London, UK; version Mascot). Mascot was set up to search the NCBInr_20090719 database (selected for *Canis lupus familiaris*, 34574 entries) assuming that digestion enzyme was non-specific. Mascot was searched with a fragment ion mass tolerance of 0.50 Da and a parent ion tolerance of 20 ppm. Iodoacetamide derivative of cysteine was specified in Mascot as a fixed modification. Deamidation of asparagine and glutamine and oxidation of methionine were specified in Mascot as variable modifications. Scaffold (version Scaffold_3_00_08, Proteome Software Inc., Portland, OR) was used to validate MS/MS based peptide and protein identifications. Peptide identifications were accepted if they could be established at greater than 95.0% probability as specified by the Peptide Prophet algorithm [35]. Protein identifications were accepted if they could be established at greater than 99.9% probability and contained at least 5 identified peptides. Protein probabilities were assigned by the Protein Prophet algorithm [36]. Proteins that contained similar peptides and could not be differentiated based on MS/MS analysis alone were grouped to satisfy the principles of parsimony.

### Western immunoblotting

Following SDS-PAGE, the separated proteins were electrotransfered at 100 V for 1 h onto Immobilon-P polyvinylidene fluoride (PVDF) membrane (Millipore, Billerica, MA). The membrane was blocked for 2 h with phosphate-buffered saline, pH7.4 containing 0.1% Tween-20 (PBS-T) and 5% non-fat skim milk. The membrane was then incubated with polyclonal rabbit anti-protein C (HPC4) tag antibody (GenScript, Piscataway, NJ) at 1:1,000 dilution (5 × 10^-4^ μg/μL) in PBS-T containing 5% non-fat skim milk for 60 min, followed by three 10-min washes in 20 mL of PBS-T. The membrane was then incubated with horseradish peroxidase (HRP)-conjugated polyclonal goat anti-rabbit IgG antibody (Dako, Denmark) at 1:2,000 dilution (1.25 × 10^-4^ μg/μL) in PBS-T containing 5% non-fat skim milk for 2 h and washed three times as described above. The membrane was then incubated with HRP conjugate for the MW standards (Precision Plus Protein StrepTactin-HRP Conjugate, Bio-Rad, Mississauga, ON) at 1:5,000 dilution (2 × 10^-4^ μg/μL) in PBS-T containing 5% non-fat skim milk for 20 min, followed by three washes as described above. The membrane was then incubated with the Amersham ECL Western Blotting Detection Reagents (GE Healthcare, Buckinghamshire, UK) according to the manufacturer’s instructions. Chemiluminesence was detected using the ChemiDoc XRS + System with Image Lab Software (BioRad, Mississauga, ON). Relative intensity of detected bands was determined by densitometry using Image Lab Software.

Additionally, the purified proteins were subjected to Western immunoblotting using a mouse monoclonal anti-human protein C heavy chain antibody, clone GMA-093 (Upstate, Lake Placid, NY), as primary antibody at 1:1,000 dilution (1 × 10^-3^ μg/μL) and a polyclonal rabbit anti-mouse IgG antibody coupled to horseradish peroxidase (Abcam, Cambridge, MA) as secondary antibody at 1: 1,000 dilution (5 × 10^-4^ μg/μL). Procedures for Western immunoblotting and detection of immunoreactive proteins were carried out in the same way as described above.

### N-linked glycosylation mapping

For visualization of deglycosylated proteins on SDS-PAGE, the isolated proteins were incubated with a Protein Deglycosylation Mix (New England Biolabs, Mississauga, ON) according to the manufacturer’s instructions. Briefly, 9 μg of purified proteins were incubated with 40 mM DTT and 0.5% SDS at 100°C for 10 min and cooled on ice for 10 s. The denatured proteins were then incubated with 50 mM sodium phosphate, pH7.5, containing 1% NP-40, O-glycosidase (4 × 10^3^ units/μL), beta-N-acetylglucosaminidase (4 × 10^-1^ units/μL), neuraminidase (5 units/μL), beta-1-4-galactosidase (8 × 10^-1^ units/μL), and PNGase F (5 × 10 units/μL) for 4 h at 37°C in a 50-μL reaction. Purified proteins subjected to the same procedures in the absence of the deglycosylation enzymes served as a negative control. Bovine fetuin (a glycosylated protein) was used as a positive control. SDS-PAGE and silver-staining were performed as described above. Proteins isolated from three different dogs were studied.

To identify potential N-linked glycosylated sites, the predicted amino acid sequence of CnPC was searched for the consensus sequences N-X-T and N-X-S [19]. For IGOT [21], 10 μg of purified protein was first concentrated and diafiltered into 10 μL of ^18^O-labelled water (97% atom labelled) (Sigma-Aldrich, Oakville, ON) using a Microcon Centrifugal Filter Device with molecular weight cut-off at 10 kDa (Millipore, Billerica, MA). Following addition of 2 μL of 400 mM DTT and 5% SDS, the proteins were denatured (total volume: 12 μL) at 100°C for 10 min and cooled on ice for 10 s. The following were then added to the mixture: 20 μL of ^18^O-labelled water, 4 μL of 500 mM sodium phosphate, pH7.5, 2 μL of 10% NP-40, and 2 μL of PNGase F (5 × 10^2^ units/μL). This mixture was incubated for 4 h at 37°C with periodic mixing. At the end of the reaction period, the mixture was diafiltered into TBS constituted with Milli-Q (mostly H_2_^16^O) water using a Microcon Centrifugal Filter Device with molecular weight cut-off at 10 kDa (Millipore, Billerica, MA). The proteins were subjected to trypsin digestion and LC-MS as described above. The software PEAKS (Bioinformatics Solutions, Waterloo, ON) was used to search for +3 mass unit shifts at the predicted N-linked glycosylated sites.

### Digestion of CnPC with Protac

Five μg of purified proteins in TBS were incubated with 0.1 unit of Protac (Sekisui Diagnostics, Stamford, CT), an activator of human [22] and canine [24] protein C and a substance derived from the venom of *Agkistrodon c. contortrix*, or same volume of TBS at 37°C for 15 min. All samples were then subjected to non-reducing 12% SDS-PAGE, followed by silver-staining or Western immunoblotting using a polyclonal rabbit anti-protein C (HPC4) tag antibody as described above.

### N-terminal sequence determination

The N-termini of the heavy chain, light chain, and Protac-digested heavy chain were analyzed by Edman degradation [37]. Purified proteins were subjected to reducing 12% SDS-PAGE and electrotransfered overnight at 30 V in CAPS buffer (Sigma-Aldrich, Oakville, ON) onto Immobilon-P^SQ^ PVDF membrane (Millipore, Billerica, MA). The membrane was incubated for 10 mins in 0.2% (w/v) Ponceau S (Sigma-Aldrich, Oakville, ON) in 1% (v/v) acetic acid and washed three times in Milli-Q water. It was then stored in a moist chamber and submitted for sequencing (The Advanced Protein Technology Centre, Hospital for Sick Children, Toronto, ON). Stained protein bands were excised and analyzed by the Procise Protein Sequencing System (Applied Biosystems, Foster City, CA) as previously described [38].

### Detection of amidolytic activity

The amidolytic activity of the purified protein was measured by a method modified from previously described studies [22-24,39] using Protac and Spectrozyme [H-D-(γ-carbobenzoxyl)-lysyl-prolyl-arginine-paranitroanilide diacetate salt], a chromogenic substrate for activated protein C. Protac (Sekisui Diagnostics, Stamford, CT) and Sepctrozyme (Sekisui Diagnostics, Stamford, CT) were reconstituted with sterile water to 0.5 unit/mL and 10 mM, respectively. Spectrozyme was further diluted by combining 7 parts of 50 mM Tri-HCl, pH 8.4 and 1 part Spectrozyme. The purified protein was adjusted to 0, 20, 40, 60, 80, and 100 μg/mL by dilution with TBS. Fifty μL of purified protein was incubated with 50 μL of Protac (0.5 unit/mL) for 5 min at 37°C. Forty μL of this mixture was placed in each well in a 96-well plate. One hundred and eighty μL of Spectrozyme was added to each of all samples simultaneously using a multi-channel pipette. Final protein concentrations in the samples were 0, 2, 4, 6, 8, and 10 μg/mL. Absorbance at λ = 405 nm was measured at 0, 1, 2, 3, 4, 5, 6, 7, 8, 9, and 10 min following addition of Spectrozyme using a BioTek Synergy HT plate reader (BioTek, Winooski, VT) and Gen5 version 1.11 (BioTek, Winooski, VT). All samples were analyzed in duplicate and the average of the two measurements was used as one true replicate for statistical analysis using GraphPad Prism (GraphPad Software, La Jolla, CA). For each concentration of protein (final concentrations: 0, 2, 4, 6, 8, 10 μg/mL), three replicates were obtained using proteins isolated from three different dogs. The mean absorbance ± standard error of mean (SEM) for each time point was plotted against time. For each time point, the mean absorbance ± SEM was plotted against concentration of purified protein. Additionally, amidolytic activities in canine plasma and the salt precipitate obtained following barium chloride and ammonium sulphate precipitation were determined in a similar manner using final protein concentration of 100 μg/mL.

### Canine factor V & VIII assays

Canine coagulation factor assays were performed based on published methods [40,41] using the KC 4 Delta coagulation analyzer (Stago, Parsipanny, NJ). Canine plasma pool was generated by pooling 0.5–2 mL of citrated plasma from each of ten clinically healthy adult dogs. The plasma pool was stored in aliquots at -70°C. For calibration, the activity level of each coagulation factor present in the undiluted plasma pool was designated to be 100% in the calibration process; and the plasma pool was serially diluted five times by a factor of two to obtain samples with activity levels of 50%, 25%, 12.5%, 6.25%, and 3.125%. The isolated canine protein was activated with Protac as described above and prepared by dilution with TriniCLOT imidazole buffer (Diagnostica Stago, Toronto, ON) to obtain these concentrations of 1, 0.8, 0.6, 0.4, 0.2 and 0 μg/μL. Fifty μL of factor V-deficient plasma (Trinity Biotech, Jamestown, NY) or factor VIII-deficient plasma (Trinity Biotech, Jamestown, NY) was incubated with 50 μL of pooled canine plasma and 3 μL of purified protein for 60 s at 37°C with constant mixing, followed by incubation with 10 μL of TriniCLOT aPTT reagent S (Diagnostica Stago, Toronto, ON) at 37°C for 300 sec. One hundred μL of 0.025 M CaCl_2_ (Diagnostica Stago, Toronto, ON) was added to the mixture and the time from then to clot formation was recorded [referred to as modified activated partial thromboplastin time (APTT)]. All samples were analyzed in duplicate and the average of the two measurements was used as one true replicate for statistical analysis using GraphPad Prism (GraphPad Software, La Jolla, CA). The clotting times were compared against a standard curve generated within the same assay using canine pooled plasma diluted with TriniCLOT imidazole buffer. Percent activity of FV or FVIII was plotted against concentration of the purified canine protein.

## Abbreviations

FV: Factor V
FVIII: Factor VIII
FDA: Food and Drug Administration
EMEA: European Medicines Agency
PROWESS: Recombinant human activated protein C worldwide evaluation in severe sepsis
CnPC: Canine protein C
CnAPC: Canine activated protein C
SDS-PAGE: Sodium dodecyl sulphate polyacrylamide gel electrophoresis
DELTA-BLAST: Domain-enhanced lookup time-accelerated BLAST
LC-MS/MS: Liquid chromatography followed by tandem mass spectral analysis
IGOT: Isotope-coded glycosylation site-specific tagging
PTM: Post-translational modification
PVDF: Polyvinylidene fluoride
PBS-T: Phosphate-buffered saline, pH 7.4 containing 0.1% Tween-20
SEM: Standard error of mean
APTT: Activated partial thromboplastin time
